# Mysterious ureteral migration of a wooden toothpick from the digestive tract: case report and diagnostic considerations

**DOI:** 10.31744/einstein_journal/2022RC5743

**Published:** 2022-01-27

**Authors:** Ricardo Di Migueli, Ricardo De La Roca, Fernando Korkes

**Affiliations:** 1 Hospital São Luiz Jabaquara Rede D’Or São Luiz São Paulo SP Brazil Hospital São Luiz Jabaquara, Rede D’Or São Luiz, São Paulo, SP, Brazil.; 2 Centro Universitário FMABC Santo André SP Brazil Centro Universitário FMABC, Santo André, SP, Brazil.

**Keywords:** Foreign bodies, Ureter, Toothpick

## Abstract

Migration of foreign bodies into the urinary tract is a rare event. In certain instances, to unravel the way that objects arrived in the urinary tract is not easy. We report the case of an accidentally swallowed wooden toothpick that migrated and was found in the left ureterovesical junction, protruding into the bladder. Even though the computed tomography scan is widely employed to evaluate the urinary tract, this resource does not have a good sensitivity for detecting foreign bodies. Our report presents an insight into the best imaging approach if wooden toothpicks are suspected. In the present case, the endoscopic treatment was possible with an uneventful outcome and a complete resolution of symptoms.

## INTRODUCTION

There are numerous reports of foreign bodies found within the cavities of various organs of the human body.^(1)^ They can be intentionally inserted as a consequence of psychiatric disturbances or as a mean of sexual stimulation.^(2)^There are also some reports of foreign bodies that migrated from one organ to another. Sometimes this migration occurs through intriguing paths.^(2,3)^

We report an unusual case of ureteral obstruction from a swallowed toothpick that migrated from the digestive tract the left ureter with protrusion into the bladder, and a discussion is provided of the best imaging approach when wooden toothpicks are suspected.

## CASE REPORT

A 54-year-old man was referred to the clinic with complaints of repeated urinary tract infections during the previous ten months, treated with several antibiotics, with no improvements. The patient did complaint of an increased voiding frequency, dysuria, and urgency with a strong voiding stream. He had been presenting abdominal pain as cramps for the last 2 years. In addition, he undergone endoscopy and a colonoscopy that demonstrated uncharacteristic reddish areas in the transverse and descending colon. His physical examination was unremarkable.

**Figure 1:**
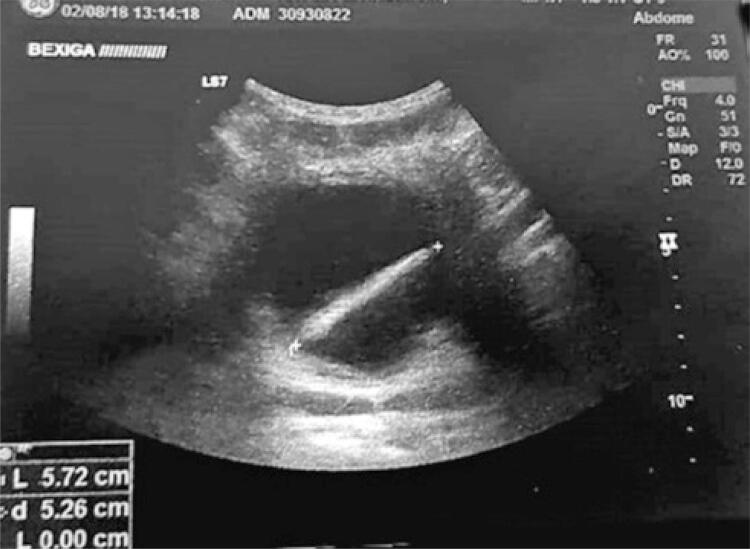
Bladder ultrasound demonstrating a 4cm linear hyperechoic image

The patient had urinary infection (leukocyturia 1x10^6^/mL and *Enterococcus faecalis* 100,000 colonies/mL). A computed tomography (CT) scan of the abdomen was performed, however, it could not find any additional abnormalities. The urodynamic study was unremarkable, except for an augmented sensibility. The patient was treated with oral cephadoxil for 10 days but the picture persisted with leukocyturia with a sterile culture (1x10^6^ leukocytes/mL and negative culture). An abdominal ultrasound was performed and demonstrated a mild dilatation of the left urinary tract with a hyperechoic image within the bladder (Figure 1).

**Figure 2:**
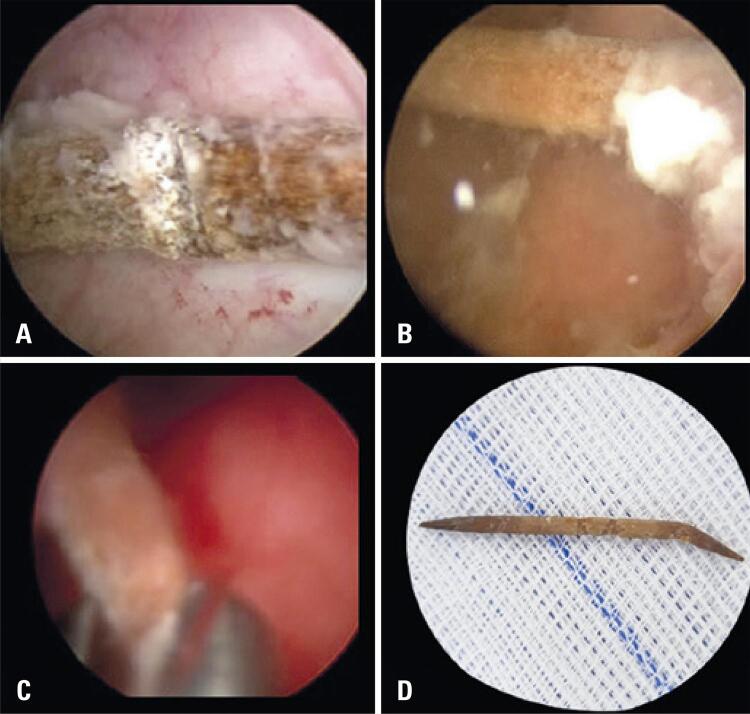
Images of the ureteral foreign body. (A) Cystoscopic view of a foreign body inside the bladder; (B) Left ureteral meatus with the protruding toothpick; (C) Cystoscopic removal of the foreign body; (D) Wooden toothpick removed from the left ureter

Subsequently, the patient underwent cystoscopy under intravenous sedation. By using a 22 French cystoscope, a brownish tubular structure with a calcified surface was visualized in the bladder. The object was emerging from the left ureteral meatus where edema and hyperemia were presented (Figure 2).

The foreign body was removed with the aid of a grasping forceps without any difficulty. After removal, the foreign body could easily be identified as a wooden toothpick. The ureteroscopy did not show any abnormalities.

Recovery was uneventful, and all urinary and abdominal symptoms resolved. Urinary tests also became normal. The patient did not recall swallowing a toothpick in the past. Besides, he assured that he had never inserted such an object through his urethra.

This study was approved by the Ethics Committee of *Rede D’Or São Luiz* (approval number: 4.278.230; CAAE: 37194220.7.0000.0087).

## DISCUSSION

Toothpick accidental ingestion is not uncommon. After ingestion, the toothpick can be eliminated spontaneously or it can cause a myriad of organ injuries.^(3)^ A systematic review performed in 2002 identified 47 articles reporting toothpicks organ injuries. As with our patient, 88% of these patients who experienced swallowing a toothpick could not remember to do so. In most cases, the injured organs were within the gastrointestinal tract.^(3)^ When foreign bodies puncture the gastrointestinal tract severe inflammatory process can occur. The most frequent sites of these perforations are ileum and jejunum (18% to 66%) while the second portion of the duodenum corresponds to 10% to 23% of the cases; perforations of the colon and sigmoid are uncommon.^(4)^ When migrating to the retroperitoneum, these foreign bodies can cause damage to the ureters or renal pelvis.^(1)^ When migrating to the peritoneal cavity, they can have serious complications such as peritonitis and acute abdomen. Their trajectory can be random, and there are reports of toothpicks lodged within the liver, vena cava, diaphragm or pericardium.^(3)^

Few reports exist on toothpicks migrating to the upper urinary tract,^(3,5-7)^ in most of them intestinal or vascular complications occurred.^(3,7,8)^ From earlier reports, it had been hypothesized that these upper tract foreign bodies resulted from retrograde insertion through the urethra. Authors believed that these foreign bodies might have found their way into the ureteral orifice, and traveled up to the ureters through reverse peristalsis.^(5)^

A review reported that 800 foreign bodies were found in the genitourinary system during more than a century of medical literature.^(8)^ Most of these foreign bodies were inserted in the urethra. The ureter was considered the rarest place to encounter these objects. Foreign bodies in the upper urinary tract are unusual. Most reports are from gunshots or iatrogenic material after gynecologic surgeries.^(6,8)^ We have found only one report of a toothpick fully migrated to the ureter and that there was a spontaneous resolution of the fistulous tract, as in our case.^(6)^

Currently, CT scans are the first line exams to evaluate upper urinary tract obstruction. Nevertheless, as occurred in our case, previously reports did not observe wooden toothpicks in CT scans.^(1)^ Indeed, only 14% of ingested toothpicks can be diagnosed through imaging exams.^(3)^ The sensibility of CT and ultrasonography to find wooden objects were estimated to be 15% and 29%, respectively.^(3)^ Plain radiography has a very low sensitivity for detecting wooden foreign bodies.^(1,3)^ In cases associated with complications as a perinephric abscess or organ injuries, the CT scan seems to be more accurate.^(9)^ A combination of ultrasonography and CT seems to be a better option to identify these objects.^(10)^ This information is crucial if, by any chance, this rare situation is suspected.

The timing of occurrence of these events can vary widely. Whereas urinary migration has been reported as soon as 24 hours after toothpick ingestion, it can also occur 15 years after the event.^(9)^ It is noteworthy that in a systematic review, a mortality rate of astonishing 18% was estimated after the ingestion of toothpicks, even though these numbers might be biased.^(3)^

As we have seen, these foreign bodies navigate within the human body through curious and unpredictable pathways. Most of symptoms can be the confounding with inflammations and infections from other causes. Imaging exams are extremely valuable for the correct diagnosis, and the combination of ultrasonography and CT should always be performed when wooden toothpicks are suspected. In the present case, cystoscopic treatment was possible and uneventful.
